# Supplementary material for: Prediction of vascular aging based on smartphone acquired PPG signals
Source: Sci Rep. 2020 Nov 12;10:19756. doi: 10.1038/s41598-020-76816-6 (PMC7661535; doi:10.1038/s41598-020-76816-6)
Supplement: Supplementary file 1 — Supplementary information. [file 41598_2020_76816_MOESM1_ESM.pdf]

Prediction of vascular ageing based on smartphone acquired PPG signals

Supplementary Materials

Lorenzo Dall'Olio, Nico Curti, Daniel Remondini, Yosef Safi Harb, Folkert W. Asselbergs, Gastone Castellani, Hae-Won Uh

1 Data description

The complete dataset is crowdsourced via a campaign (Heart for Heart) and data collection is performed via a dedicated application (Happitech app). The collected data consists of two parts: general information about the user and measurement. For each one of them, we have the PPG signal for the R, G, B channels of the camera sensor, each frame's time-stamp and the values of the accelerometer along three orthogonal axes X, Y, and Z. All of these values are in arbitrary units, apart from the time, which is measured in milliseconds. Frame rate per second is 30 fps and each acquisition lasts about 90 seconds. The user was guided by the app in placing his or her fingertip upon the camera of the smartphone. The LED flash would then illuminate the finger for the entirety of the data acquisition process, which must be performed at rest (see Figure S1).

The general information part contains information about the user. A unique anonymous ID (not traceable to the original user) is also associated to each subject. Each user has also voluntarily provided additional information: age, sex, weight, height, location (city), any known heart condition, lifestyle, and smoking. Since these are self-reported measures, many missing values were present and the reliability of the data could not be verified (Bussola, 2019).

Therefore, for the current analysis, we selected five variables with the least missing values: age, sex, height, weight, and smoking status.

2 PPG Preprocessing

The python code for preprocessing can be found on GitHub, `https://github.com/Nico-Curti/cardio`. An accurate representation of the code for a single signal is given as follows:

- We start with a raw signal raw consisting of the acquisition times sequence raw_{Time} (Figure S2), and 3 time series (respectively the red raw_R, green raw_G and blue raw_B reflected light components), with all of the 4 sequences of equal length.
- Since the emitted light came from a white flash, all the 3 light components should carry approximately the same information rescaled by light absorbance, and so with the only difference in Signal to noise ratio, due to the fact that some wavelengths are more absorbed by the tissues. Therefore we keep only the red component (raw_R), since it is the one with higher amplitude and better signal to noise ratio.
- We compute the average sampling frequency sf as

$$sf = \frac{\text{Nr. points in } raw_{Time}}{\max(raw_{Time}) - \min(raw_{Time})} \tag{1}$$

  where the usage of $\min(raw_{Time})$ is needed, because the acquisitions do not begin exactly at $Time = 0$.
- We compute the half-width w of the window that we will further use for the central moving average as

$$w = \lfloor \frac{sf}{2} \rfloor \tag{2}$$

where $\lfloor\cdot\rfloor$ is the floor rounding operator, and the division by 2 is in reality a multiplication times 0.5 (because multiplications are computationally faster than divisions). Doing so, we will approximately consider $2w + 1$ points in the computation of the central moving average, and recalling that $w = \lfloor sf/2 \rfloor$ we obtain that the number of points considered for the moving average is $\approx sf$ for $sf > 10$. In this way we have a total time width for our moving average of ≈ 1 second, and since for our data usually $sf \approx 30Hz$ the approximation can be considered as reliable.

- At this point we compute the CMA by using a "trick", in fact we achieve such result by convolving a box of $2w + 1$ width and unitary area with the red component raw_R, giving us

$$\mathrm{CMA}(raw_R, w) = raw_R * \frac{\overbrace{[1, 1, 1, \ldots, 1, 1]}^{2w+1 \text{ elements}}}{2w+1}. \tag{3}$$

  Thanks to this, we achieve a faster and vectorized way to perform

$$\mathrm{CMA}(raw_R)_i = \frac{\sum_{i=-w}^{+w} raw_R}{2w+1}. \tag{4}$$

  Please notice that the *total* width of the moving average is given by $2w + 1$, which is always an odd number since w is an integer. This fact allows us to always have a unique central point for the window, which is what guarantees that we are performing a *central* moving average. We want to clarify that the boundary conditions are treated in a "valid" mode, which means that we will discard any point that will not have at least w points before and w points after it.

- As above explained, the CMA computation involves the removal of $2w$ points, the first w and the last w, from both raw_R and raw_{Time} as this points are ignored in the computation of the CMA

$$raw_{R,cut} = raw_R[w : -w] \tag{5}$$
$$raw_{Time,cut} = raw_{Time}[w : -w]. \tag{6}$$

- At this point, having the lower frequency components in the computed CMA, to obtain a high pass filter we simply subtract it from the original raw component. This gives us the detrended signal

$$detrended_R = raw_{R,cut} - CMA(raw_R, w) \tag{7}$$
$$detrended_{Time} = raw_{Time,cut}. \tag{8}$$

- now we can compute the analytic signal from the detrended signal using

$$analytic = detrended_R + i\mathcal{H}(detrended_R) \tag{9}$$

  where $\mathcal{H}(detrended_R)$ indicates the Hilbert transform. Please notice that on the used python library (*scipy*) the function *scipy.signal.hilbert(x)* does not compute the Hilbert transform, but directly the analytic signal.

- Now we have the analytic signal, this is a complex-valued signal, so each of its elements will be characterized by an amplitude and a phase. Computing the absolute value of the analytical signal we recover the instantaneous amplitude, also called *envelope*, of the signal

$$envelope = |analytic| \tag{10}$$

- None of the previous operation has further shortened the arrays, therefore we compute the new average sampling frequency as

$$sf_{cut} = \frac{\text{Nr. points in } raw_{Time,cut}}{\max(raw_{Time,cut}) - \min(raw_{Time,cut})} \tag{11}$$

  .

- Now we will smooth the envelope in order to remove very high frequencies (denoising) by computing its CMA with half-width of $w_{env} = \lfloor sf_{cut} \rfloor$ as a low pass filter, hence

$$\mathrm{CMA}(envelope, w_{env}) = envelope * \frac{\overbrace{[1,1,1,\ldots,1,1]}^{2w_{env}+1 \text{ elements}}}{2w_{env}+1} \tag{12}$$

  and again this will imply a shortening in the signal of $2w_{env}$ points, half at the begin and half at the end, for both $detrended_R$ and $raw_{Time,cut}$ (we are not considering *envelope* because we will no further use it, but if we needed to use it we would have to short it too)

$$detrended_{R,second\ cut} = detrended_R[w_{env} : -w_{env}] \tag{13}$$

$$raw_{Time,second\ cut} = raw_{Time,cut}[w_{env} : -w_{env}]. \tag{14}$$

- The last step consists in dividing the the detrended shortened signal by the smoothed envelope to perform demodulation

$$demoduled_R = \frac{detrended_{R,second\ cut}}{\mathrm{CMA}(envelope, w_{env})}. \tag{15}$$

- The preprocessing algorithm concludes by returning two arrays: $demoduled_R$ (which has also been detrended and denoised) and $raw_{Time,second\ cut}$. These tow arrays are exactly $2w{+}2w_{env}{+}2$ points shorter than the input arrays, and consist the time series which will be analyzed and which will be used for further features extraction.

Figure S3 depicts a part of the peak detection algorithm regarding a portion of a processed signal, resulting in Fig. 2 in the main paper: the raw (top), detrended (middle), demoduled and denoised (bottom) signal.

3 The list of 38 features extracted from the PPG signals

- SDPPG group:
  - ***a***, ***b***, ***c***, ***d***, ***e*** (see Figure S4), ***ab_ slope*** (slope of the segment connecting point ***a*** and point ***b*** in the SDPPG), ***ac_ slope***, ***ad_ slope***, ***ae_ slope***, ***bc_ slope***, ***bd_ slope***, ***be_ slope***, ***cd_ slope***, ***ce_ slope***, ***de_ slope***, ***t_ ab*** (time interval that separates point ***a*** and point ***b*** in the SDPPG), ***t_ ac***, ***t_ ad***, ***t_ ae***, ***t_ bc***, ***t_ bd***, ***t_ be***, ***t_ cd***, ***t_ ce***, ***t_ de***.
- PPG group:
  - Extracted from RR: ***ibi*** (average inter beat interval, basically the mean of RR), ***medianRR***, ***entropyRR***, ***kurtosisRR***, ***skewnessRR***, ***madRR*** (median absolute distance of RR), ***tpr*** (turning point ratio of RR, basically it is number of local extrema in RR divided by the number of elements in RR), ***sdnn*** (standard deviation normal to normal, basically standard deviation of RR).
  - Extracted from RR_{diff}: ***pnn20*** (proportion of normal to normal 20, the percentage of elements of RR which differ of more than 20 ms from the successive element, basically the percentage of elements of RR_{diff} greater than 20 ms), ***pnn50***, ***sdsd*** (standard deviation of successive differences), ***rmssd*** (root mean square of successive differences), ***tpr_ RR_ diff***.

4 Regression analysis using the continuous outcome, age.

In our work, we considered the classification task of healthy or unhealthy vascular aging, which can be smoothly transferred to any disease outcome. If one's choice is to accurately predict continuous outcome, such as age or blood pressure, we can perform a regression analysis. For non-parametric DL, CNN was modified as follows:

- Consider the best performing CNN from previous analysis

- Change the output layer to 1 single neuron (which will be used for regression against the chronological age)
- Modify the loss function from categorical cross-entropy to Mean Absolute Error (MAE), which was the best performing among all other regression loss functions.
- Train until no further improvement in the loss function was observed for more than 10 epochs.

This procedure resulted in a training of around 100 epochs and a performance on the test set through a linear regression reported in Figure S6. Further we performed a linear regression and a ridge regression with inner cross validation for the penalization parameter, both trained on the training set and evaluated on the test (see Figures S8 and S7).

We compared ML and DL results using R^2 and Mean Squared Error (MSE) as metrics. Note that, since the DL CNN was working on slices of 15 consecutive PPG peaks, the number of points in the DL plot is higher, but the portion of dataset used for comparison is exactly the same (the test set).

References

Bussola, F. Quantitative analysis of smartphone PPG data for heart monitoring. `https://amslaurea.unibo.it/id/eprint/18150` (2019)

Uh, H.W. Prediction of vascular aging based on smartphone acquired PPG signals. IBC 2020 conference recording. `https://www.dropbox.com/s/4a09zvrd8dsi57o/Hae-Won%20Uh_Session8.mp4?dl=0` (2020)

Tables and Figures

Model	Sensitivity (%)	Specificity (%)	AUC (%)
38 features + covariates	87.76	85.42	95.43
CNN: 12-layers-ResNet	90.72	85.83	95.34
a + tpr + covariate	87.34	86.25	94.73
ac_slope + tpr + covariates	87.76	88.33	94.60
a + tpr	83.97	82.50	87.43
ac_slope	78.06	78.75	81.68
a	77.64	78.33	80.79
tpr	73.00	76.25	80.50
covariates	69.62	72.50	74.21
pnn20	66.67	61.67	66.29
ibi	40.93	72.50	55.21

Table S 1: Sensitivity, Specificity and AUC scores for some of the compared models.

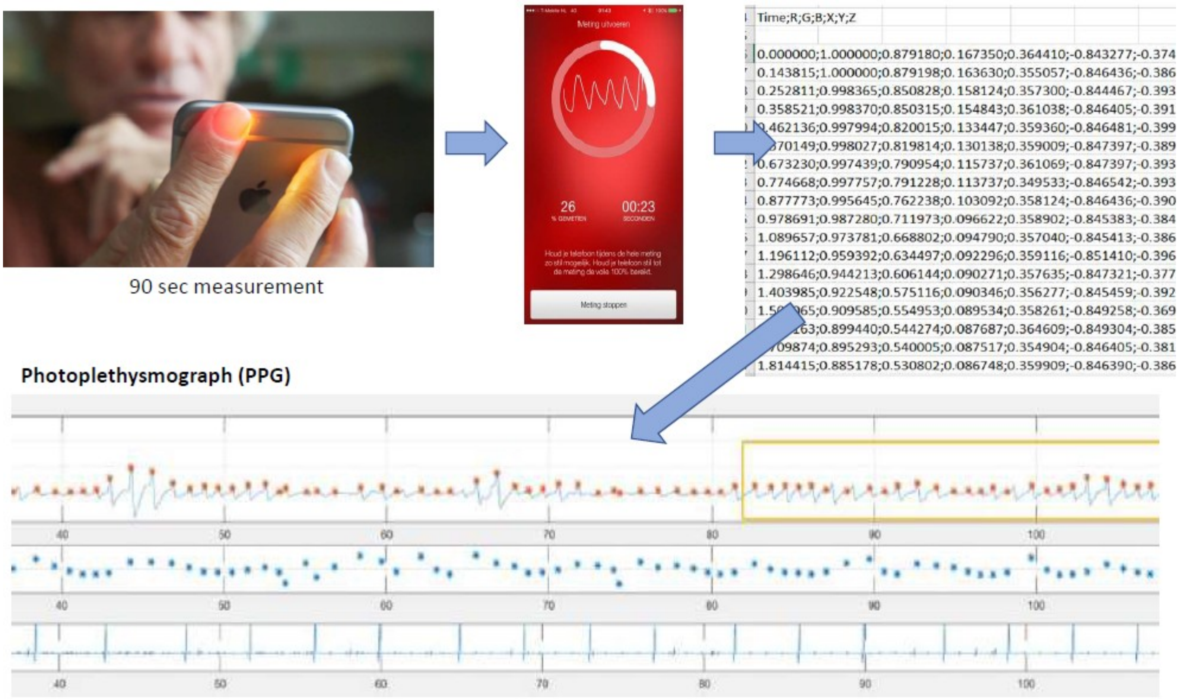

Figure S 1: Data capture (Source: Hae-Won Uh, 2020).

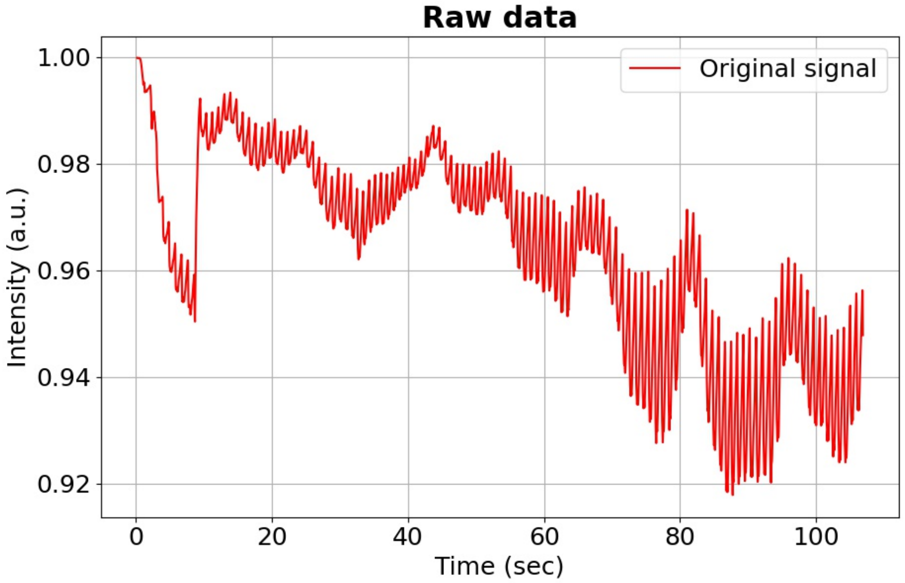

Figure S 2: Raw signal (90 seconds, 30 frame rate per second).

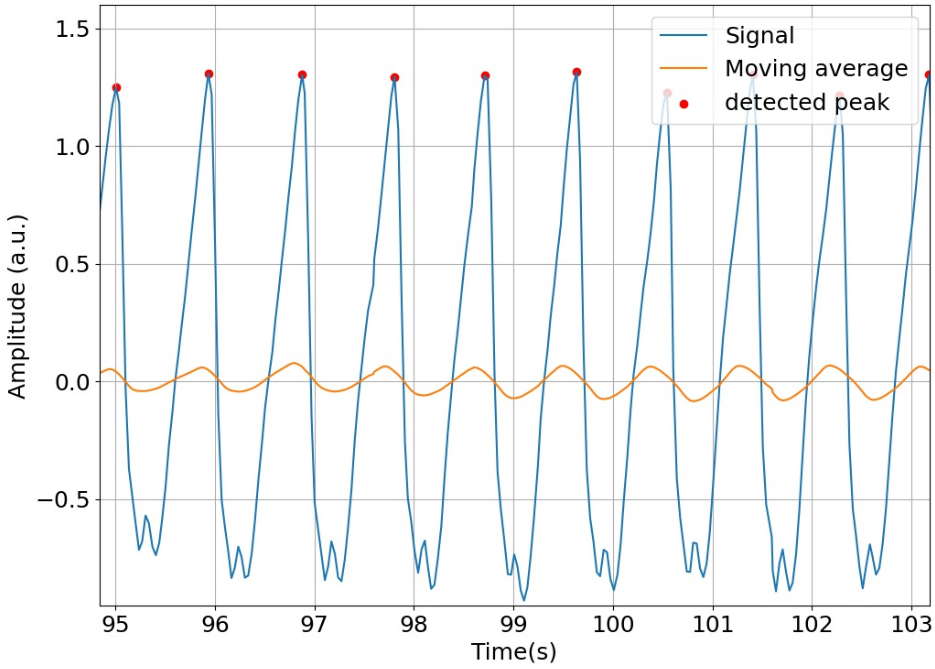

Figure S 3: Peak detection snapshot, using a CMA's window width of 1 period. note that every detected peak displays a reasonably correct detection.

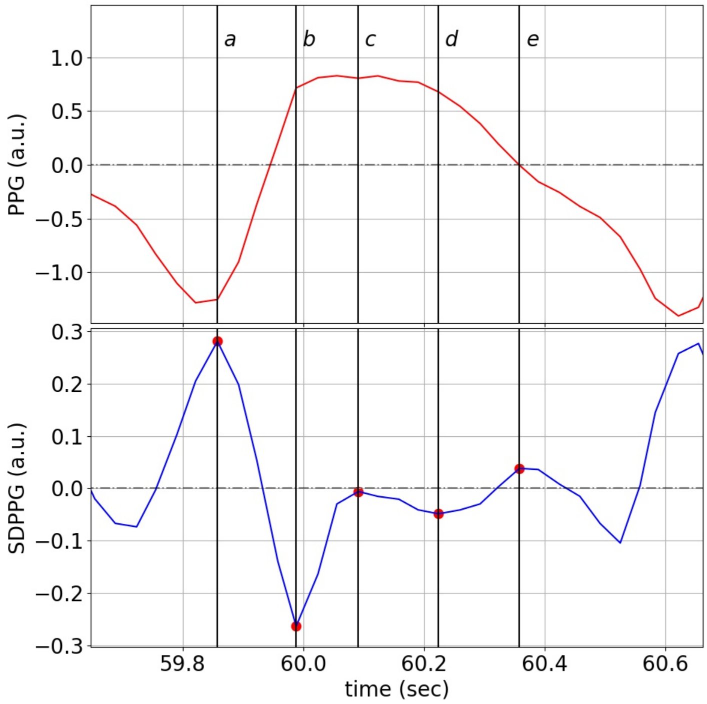

Figure S 4: Comparison of PPG (top) and SDPPG (bottom) shapes. Red points represent the peaks and changing points, ***a***, ***b***, ***c***, ***d*** and ***e***, identified by SDPPG.

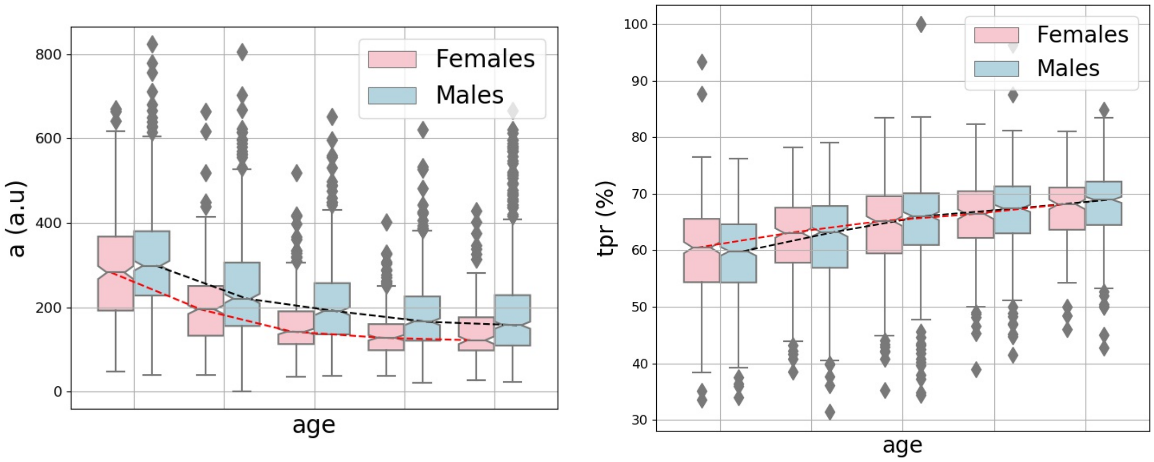

Figure S 5: Boxplots of age quintiles stratified by sex. Please note that ***tpr*** (right) increases with age, while ***a*** (left) decreases and is appreciably lower for females.

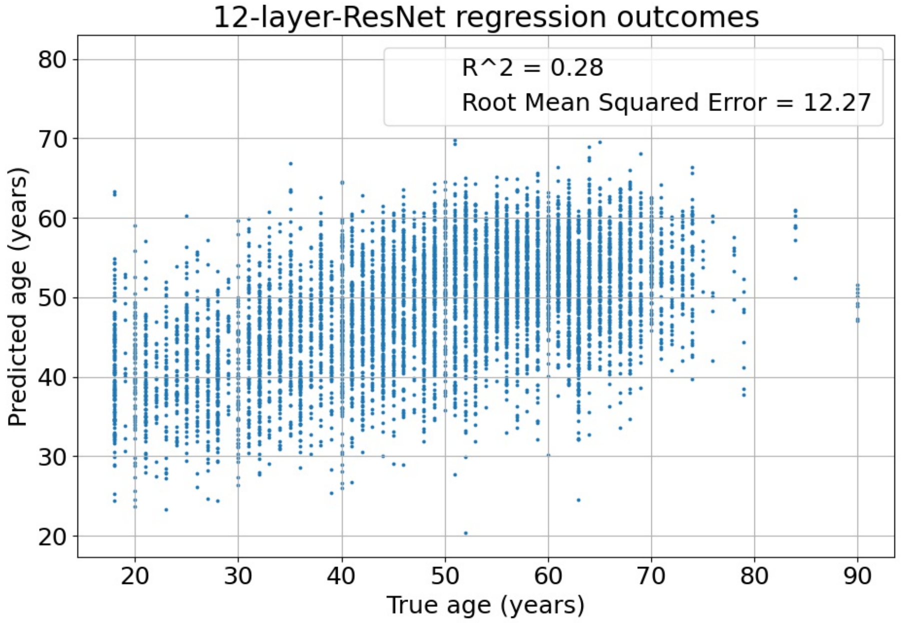

Figure S 6: Results of DL on continuous age: True age (x-axis) vs. Predicted age (y-axis)

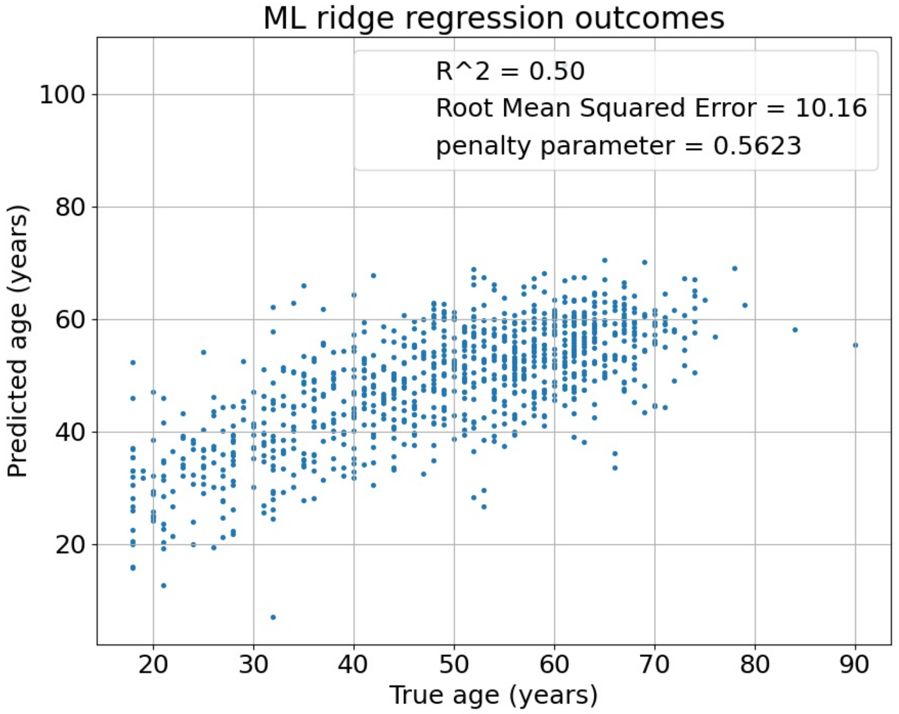

Figure S 7: Results of ridge penalized regression on continuous age: True age (x-axis) vs. Predicted age (y-axis). Here, the predictors were 38 extracted features and four additional covariates. The optimal penalization parameter was 0.5623.

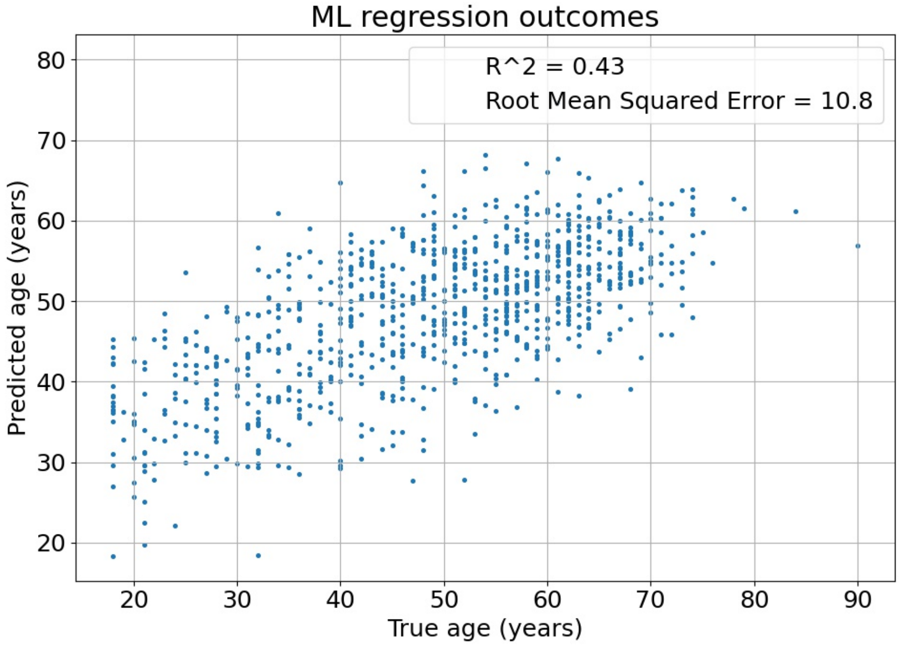

Figure S 8: Linear regression outcomes using $\boldsymbol{a}$, $\boldsymbol{tpr}$ and four covariates as predictors.
